# The enigma of optical momentum in a medium

**DOI:** 10.1098/rsta.2009.0207

**Published:** 2010-03-13

**Authors:** Stephen M. Barnett, Rodney Loudon

**Affiliations:** 1 Department of Physics, SUPA, University of Strathclyde, Glasgow G4 0NG, UK; 2 School of Computer Science and Electronic Engineering, University of Essex, Colchester CO4 3SQ, UK

**Keywords:** Abraham–Minkowski dilemma, photon momentum, Poynting vector, quantum optics

## Abstract

It is 100 years since Minkowski and Abraham first gave rival expressions for the momentum of light in a material medium. At the single-photon level, these correspond, respectively, either to multiplying or dividing the free-space value (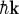) by the refractive index (*n*). The debate that this work started has continued till the present day, punctuated by the occasional publication of ‘decisive’ experimental demonstrations supporting one or other of these values. We review the compelling arguments made in support of the Minkowski and Abraham forms and are led to the conclusion that *both* momenta are correct. We explain why two distinct momenta are needed to describe light in a medium and why each appears as the natural, and experimentally observed, momentum in appropriate situations.

## 1. Introduction: the Abraham–Minkowski dilemma

It has long been appreciated that light has mechanical properties. Indeed, Maxwell (1891) presented a simple calculation of the pressure exerted by sunlight at the surface of the Earth. It was Poynting (1884) who determined that it is the cross-product of the electric and magnetic fields that determines the flux of electromagnetic energy. For light propagation in vacuum, there is no difficulty in also identifying this cross-product with the density of electromagnetic momentum. Within a medium, however, we have a choice to make between the electric and displacement fields (**E** and **D**) and the magnetic field and the magnetic induction (**H** and **B**). Poynting’s theorem tells us that the flux of energy is **E**×**H**, but there are two entirely reasonable and rival forms for the corresponding density of momentum. These are the Minkowski (1908) momentum density, **g**_Min_=**D**×**B** and the Abraham (1909, 1910) momentum density, **g**_Abr_=**E**×**H**/*c*^2^. The problem of determining which momentum is ‘correct’ is the famous Abraham–Minkowski dilemma. This is not the place to review the large literature devoted to this problem; instead, we recommend to the interested reader the review by Brevik (1979) and the more recent one of Pfeifer *et al.* (2007).

It is not necessary to quantize the electromagnetic field in order to appreciate the problem, but it is helpful to understand it in terms of the properties of a single photon of angular frequency *ω*. We can do this by means of a simple scaling argument. The total electromagnetic energy within our volume is simply that of the photon (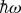):
1.1
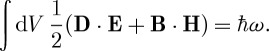

This energy is (on average) shared equally between the electric and magnetic parts so that
1.2
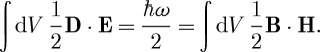

If we consider, for simplicity, a linear isotropic and homogeneous medium with relative permittivity *ε* and permeability *μ*, then we are led to
1.3
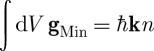

and
1.4
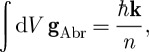

where **k** is the wavevector in vacuum (with magnitude *ω*/*c*) and 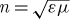 is the refractive index of the medium. At its simplest, therefore, Minkowski would assert that the momentum of a photon in a medium is its value in vacuum *multiplied* by the refractive index, while Abraham would have us believe that it is the vacuum value *divided* by the refractive index. In dispersive media, the situation is a bit more complicated in that we need to discriminate between phase and group indices (Garrison & Chiao 2004; Loudon *et al.* 2005; Milonni & Boyd 2005; Bradshaw *et al*. in press), but, in the interests of simplicity, we shall leave this feature until §6.

### (a) Argument in favour of Abraham

Perhaps the most direct way to calculate the momentum of a photon in a medium is to use the Newtonian idea that the centre of mass (or more precisely the centre of mass-energy) of an isolated system undergoes uniform motion (Einstein 1906). We follow the analysis of Balazs (1953) and apply this idea to a single photon and a block of transparent material initially at rest. We let the photon travel in the *z*-direction and are then interested in this component of the electromagnetic momentum. The photon has energy 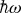 and propagates with speed *c*. If the block has mass *M* then the total energy is
1.5
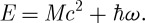

When the photon enters the medium, its speed slows to *c*/*n* and, as a result, it takes the time *T*=*nL*/*c* to travel through the medium, where *L* is the thickness of the block. It follows that, on leaving the block, the photon has travelled a distance (*n*−1)*L* less than it would have done had it been travelling in vacuum. This deviation from uniform motion can only be made up if the block itself was displaced in the direction of propagation of the photon by an amount Δ*z*, while the photon was in the medium. The centre of mass-energy moves uniformly if (Frisch 1965)
1.6
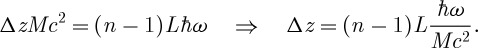

We see clearly that this displacement depends simply on the thickness of the medium, the ratio of the photon and medium energies, and the refractive index.

In order to move the distance Δ*z* while the photon is in the medium, the block must have acquired from the photon the momentum
1.7
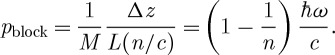

Global conservation of momentum then dictates that the total momentum is 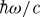 and hence that
1.8
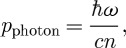

which is the value obtained by Abraham’s prescription.

We have used only the conservation of momentum and the uniform motion of the centre of mass-energy in deriving our result, and it is difficult to see how any component of our derivation could seriously be open to question.

### (b) Argument in favour of Minkowski

The first thing to be said in support of the Minkowski momentum is that it is ‘natural’ in that the wavevector in a medium is greater than that in vacuum by the refractive index and hence the Minkowski single-photon momentum (1.3) is simply 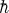 multiplied by the wavevector in the medium. There are also, however, at least two simple physical arguments in support of the Minkowski momentum.

The first, due to Padgett (2008), is based on single-slit diffraction. A plane wave propagating in the *z*-direction towards a single slit in the *x*–*y* plane will undergo diffraction and produce a characteristic interference pattern in the far field. We can determine the width of the central peak of this pattern by a simple application of the Heisenberg uncertainty principle. If the slit has width Δ*x* then the uncertainty principle requires that the field after the slit has a spread of momenta in the *x*-direction of 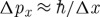. It then follows that the angular spread of the central interference peak will be
1.9
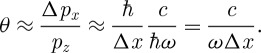

If we repeat the experiment in a medium of refractive index *n*, then we find that the angular width of the peak is *reduced* by *n*. The momentum width Δ*p*_*x*_ is imposed by the width of the slit, so this reduction can only arise because the momentum in the *z*-direction is *increased* by *n*,
1.10
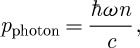

which is the Minkowski momentum. A similar result can be obtained with reference to double-slit diffraction (Brevik 1981).

Our second argument (Bradshaw *et al*. in press) is a variant of an idea due to Fermi (1932). Consider an atom of mass *m* with a transition at angular frequency *ω*_0_. Let the atom be in a medium with refractive index *n* and moving with velocity *v* away from a source of light with angular frequency *ω*. The atom can absorb a photon from the beam if the Doppler-shifted frequency matches the transition frequency, so that
1.11
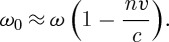

Let *v*′ denote the velocity of the atom after it has absorbed the photon. The conservation of energy and of momentum then require that
1.12
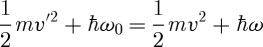

and
1.13
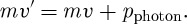

Solving these for the photon momentum gives
1.14
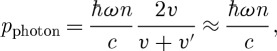

where we have made use of the fact that the absorption makes only a small change to the velocity of the atom. Simple conservation laws have led us to conclude that the photon momentum is that given by Minkowski.

These arguments in support of the Minkowski momentum are of a different character from that made in support of the Abraham form, but they are no less convincing for that. Both forms are well supported, therefore, and hence we have a dilemma.

## 2. Experimental evidence

As theory has presented us with a dilemma, it is reasonable to seek an answer in experiments, and this idea has been pursued on a number of occasions (Jones & Richards 1954; Ashkin & Dziedzic 1973; Walker *et al.* 1975; Jones & Leslie 1978). The work of Jones, Richards and Leslie confirmed that the force exerted on a mirror submerged in a medium was consistent with each photon in that medium having the Minkowski momentum. The experiment of Ashkin and Dziedzic showed that the action of light on the surface of a liquid was also consistent with the Minkowski momentum, although this interpretation is far from unambiguous (Gordon 1973). The experiments of Walker *et al.* provide evidence that is no less convincing in favour of the Abraham form. These early experiments and the conclusions derived from them are discussed at greater length in Brevik (1979) and Pfeifer *et al.* (2007).

The confusing experimental situation has continued, with further experiments seeming to support either the Minkowski or the Abraham momentum. Gibson *et al.* (1980) exploited the photon drag effect to measure the momentum transfer from far-infrared radiation to free charge carriers in germanium and silicon. In each case the observations were consistent with the Minkowski form of the optical momentum. Campbell *et al.* (2005) measured the recoil momentum of atoms in a dilute ultra-cold gas, effectively performing the experiment outlined in §1*b* above. They also found a recoil consistent with the Minkowski form. Most recently, She *et al.* (2008) measured the displacement of an optical fibre due to light leaving. Their results, although not uncontroversial (Mansuripur 2009), seem to support the Abraham momentum.

There is an angular-momentum version of the Abraham–Minkowski dilemma, with the Abraham angular momentum being that in free space divided by *n*^2^ and the Minkowski form being the same as that in free space. An angular version of the argument, given above, in support of the Abraham momentum supports, naturally enough, the Abraham angular momentum (Padgett *et al.* 2003). An experiment of Kristensen & Woerdman (1994), however, measured the torque on an object in a dielectric medium. The observations gave results in support of the Minkowski angular momentum.

Experimental work has served to confirm that the force exerted by light on an object within a medium is consistent with the Minkowski momentum for the light in that medium. The evidence in support of the Abraham momentum is, perhaps, less convincing, but tends to support the idea that the net effect on a medium due to light passing through it is consistent with the Abraham momentum. Indeed, it could not be otherwise! If the argument advanced in §1*a* in favour of the Abraham momentum were to be incorrect, then that would bring into question uniform motion of an isolated body as expressed in Newton’s first law of motion. Similarly, a failure of the arguments advanced in §1*b* in favour of the Minkowski momentum would require us to question the uncertainty principle, momentum conservation and the Doppler effect.

It seems that there is at least some validity to *both* the Minkowski and the Abraham momenta, and it is for theory to explain the origins of these two momenta and to explain why one of them appears as the ‘correct’ momentum in a specific situation.

## 3. Electromagnetic force

Our first task is to find a reliable way of determining the momentum exchange between electromagnetic fields and a medium. Using energy and momentum densities has proven to be an unreliable method for this, not least because Minkowski and Abraham have given us different expressions for the electromagnetic energy–momentum tensor. It is safer, therefore, to work with the force exerted on the medium and then to use Newton’s second law of motion to relate this to a rate of change of momentum (Loudon 2002, 2003).

The force exerted on a point dipole, with dipole moment **d**, is simply
3.1
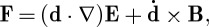

which follows directly from the Lorentz force law. It follows that the force density on a dielectric (non-magnetic) medium is (Gordon 1973)
3.2
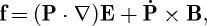

where **P** is the polarization of the medium. This is not the only possible form for the force density (Mansuripur 2004), but it has been shown that the total force exerted is the same for all acceptable choices of this density (Barnett & Loudon 2006). The force density has been used to calculate the forces exerted on dielectric media in a variety of arrangements (Loudon & Barnett 2006) but we concentrate here on the calculations relevant to photon drag experiments (Loudon *et al.* 2005).

Photon drag occurs in semiconductors and the experiments of interest were performed in silicon and germanium. At the long wavelengths used, these behave, to a good approximation, as free carriers in a background dielectric. We can safely assume that the carriers are responsible for the absorption and make a purely imaginary contribution to the permittivity. The host material is responsible for the real part. We shall assume that the medium is thick enough for our photon to be absorbed.

We consider a single-photon pulse with a narrow band of frequencies centred on *ω*. The momentum transfer to the medium is readily calculated from the force density formed from equation (3.2) by quantizing the fields and integrating over space and time. We omit the details of this calculation, which can be found in Loudon *et al.* (2005), and concentrate instead on the results and their physical significance.

The calculated momentum transfer to the free carriers is
3.3
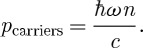

This value agrees with that found in experiments (Gibson *et al.* 1980) and also coincides with the Minkowski momentum. The calculated momentum transfer to the host material is similarly
3.4
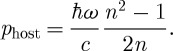

This momentum was not observable in the experiments. Adding these two gives a value for the total momentum
3.5
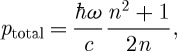

which is the mean of the Minkowski and Abraham momenta. It is straightforward to show that this value is precisely that required by global momentum conservation. A photon incident on the medium, with momentum 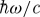, will be reflected with probability (*n*−1)^2^/(*n*+1)^2^ and transmitted into the medium with probability 4*n*/(*n*+1)^2^. Momentum conservation then requires that
3.6
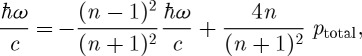

the solution of which is equation (3.5).

We can also apply our method to study a weakly absorbing dielectric with *n*≈1. If the medium is of finite thickness then this situation coincides with that proposed in §1*a* in support of the Abraham momentum. We can neglect reflections at the interfaces because *n*≈1. Our analysis confirms that the momentum transferred to the medium during propagation of a photon through it is
3.7
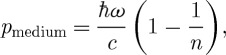

from which we can infer that the momentum of the photon in the medium is
3.8
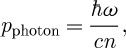

in agreement with the Abraham momentum. We note that equation (3.7) also follows directly from equation (3.4) when *n*≈1.

It is clear that the experimental observations to date are consistent with *both* the Minkowski and Abraham momenta. The momentum transfer to a body (in our case the charge carriers) within a medium is given by the Minkowski momentum. The momentum of a photon travelling through a host dielectric, however, is given by the Abraham momentum. These results have both arisen from applying the same Lorentz force to a simple model dielectric, and confirms the validity of both mechanical arguments proposed in §1*a*,*b*. It only remains to explain why there are two momenta and why they appear where they do.

## 4. The two momenta

We are not often aware of it, but we define our momentum by means of two properties. The first, which would have been familiar to Newton, is the inertial property derived from Newton’s second law of motion. This *kinetic momentum* is the product of the mass and velocity of a body. The second is most readily appreciated with reference to quantum theory as that associated with de Broglie waves. The *canonical momentum* for a quantum particle is Planck’s constant divided by its de Broglie wavelength. More formally, the canonical momentum is that derived from the Lagrangian, which is constructed to satisfy the canonical commutation relation
4.1
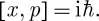

For many applications, these momenta are one and the same, but in electromagnetism they are quite distinct. The difference can be traced to the fact that the electric and magnetic fields depend on the frame of reference.

The form of the canonical momentum depends, in fact, on the gauge and the form of the matter–field coupling employed (Power 1964; Craig & Thirunamachandran 1998). In the electric dipole form, most appropriate for our systems, we find that for a single point dipole (Baxter *et al.* 1993; Leonhardt 2006; Hinds & Barnett 2009)
4.2
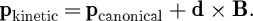

This difference arises from the Röntgen interaction, which is a manifestation of the electric field derived from a magnetic field in a moving frame of reference (in this case, that of the dipole). More generally, for an object with electric dipole moment **d** and magnetic dipole moment **m**, we find (Hinds & Barnett 2009)
4.3
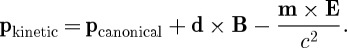

If we add together the momenta of all the dipoles in our medium, then we find
4.4
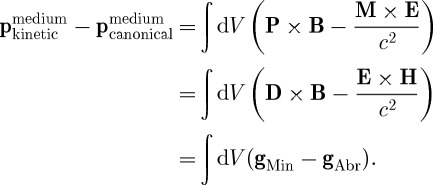

We note that the total momentum is the same, whether we are speaking of the kinetic or the canonical momentum:
4.5
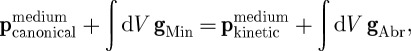

and, moreover, this total momentum is a conserved quantity.

Clearly, we can identify the Abraham momentum as the kinetic momentum of the light in the medium, while the Minkowski momentum is its canonical momentum.

## 5. A dilemma resolved: the two natural momenta

We have determined that the Abraham momentum is the kinetic momentum of the light and that the Minkowski momentum is its canonical momentum. It only remains to show how these identifications determine the conditions under which either one of them is the natural momentum.

### (a) The Abraham or kinetic momentum

The argument put forward in §1*a* depends on the displacement of a medium due to the propagation of a photon through it. This displacement is a consequence of a velocity imparted to the medium and hence a kinetic momentum. Global *kinetic* momentum conservation leads us to the kinetic momentum of the photon, which is, as we have seen, the Abraham momentum.

### (b) The Minkowski or canonical momentum

We proposed, in §1*b*, three arguments in support of the Minkowski momentum: that it is natural, that it was suggested by diffraction and that it is required by momentum conservation for absorption by a moving atom. We address each of these in turn.

It will be recalled that de Broglie identified the wavelength of a quantum body as *h*/*p*. This, in turn, led Schrödinger to express the momentum as
5.1
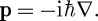

We recognize this as the canonical momentum and it is natural, therefore, that the canonical momentum, and therefore the Minkowski momentum, should be *h*/λ, where λ is the wavelength in the medium.

Padgett’s diffraction argument is based on the Heisenberg uncertainty principle and hence on the canonical commutation relation (4.1). It is for this reason that the canonical, or Minkowski, momentum is the one that appears. Equivalently, we can simply note that diffraction depends on the wavelength of the light and that this, as noted above, is inversely proportional to the Minkowski momentum.

In order to address the momentum of a body immersed in a dielectric host, we first recall the role of the canonical momentum in generating translations. The commutation relation (4.1) implies that
5.2
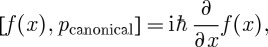

which means, in turn, that the canonical momentum is the generator of translations. The mathematical statement of this is the unitary transformation
5.3
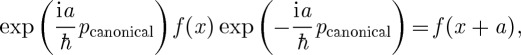

where *a* is a constant.

In the same way we find that the Minkowski momentum generates translations of the electromagnetic field in that
5.4
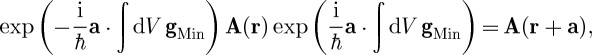

where **A** is the vector potential in the Coulomb gauge and **a** is a constant vector. For a body immersed in our medium it is precisely this translation, relative to the medium, that is important. It is for this reason that it is the canonical or Minkowski momentum that appears.

## 6. Dispersion: a final detail

One remaining issue is the forms of the two momenta in a dispersive medium, in which there are two refractive indices: the phase index
6.1
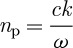

and the group index
6.2
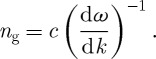

It is straightforward to show that the single-photon Abraham momentum is (Garrison & Chiao 2004; Loudon *et al.* 2005)
6.3
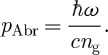

The group index, of course, determines the speed at which the photon propagates through the medium. Our argument in support of the Abraham momentum relies on this speed, and so it is entirely reasonable that it is the group index that appears. A careful calculation based on the Lorentz force law confirms this (Loudon *et al.* 2005).

The wavevector has magnitude *n*_p_*ω*/*c*, and it is reasonable, therefore, to expect that the canonical or Minkowski momentum should have the value
6.4
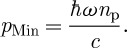

This conclusion is also supported by a calculation based on the Lorentz force (Loudon *et al.* 2005), which shows that this is the momentum transferred to a body immersed in the medium. There is a problem, however, with this value for the Minkowski momentum as
6.5
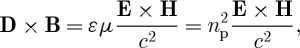

and this suggests that if equation (6.3) is the Abraham momentum then the Minkowski momentum should be 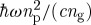 (Garrison & Chiao 2004; Milonni & Boyd 2005).

The resolution of this puzzle lies in the roles of the kinetic and canonical momenta. We can meaningfully evaluate the kinetic or Abraham momentum by taking the expectation value of **g**_Abr_. The role of the canonical momentum is to generate translations, however, and its important property, therefore, is the translation relation (5.3) or, equivalently, the commutation relation
6.6
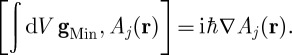

Quantization of the field inside a medium produces polaritons and, importantly, a dispersion curve with more than one frequency for each wavevector (Kittel 1987). *Each* of these branches contributes to this commutation relation by its own value of *n*_p_/*n*_g_. The total for each value of *k*, however, is (Huttner *et al.* 1991; Huttner & Barnett 1992)
6.7
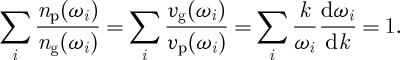

The Minkowski momentum is the canonical momentum, 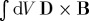, but its single-photon value is determined by the spatial shift of the field rather than by its single-photon expectation value. The Abraham and Minkowski momenta in a dispersive medium are, indeed, as given in equations (6.3) and (6.4).

## 7. Conclusion

We have described the two rival momentum densities for the light in a medium and presented, as simply as possible, the compelling physical arguments in favour of each of them. Exisiting experimental evidence strongly supports the Minkowski momentum as that transfered to a body within a host medium. There is also experimental evidence, perhaps not quite so strong, in support of the Abraham momentum as that part of the momentum not transferred to the medium during the passage of the photon through it. Calculations based on the Lorentz force reveal circumstances in which either momentum is the appropriate one and, importantly, verify the validity of the simple arguments made in favour of both the Abraham and Minkowski momenta.

The resolution of the Abraham–Minkowski dilemma lies in the realization that electromagnetism recognizes two distinct momenta, the kinetic momentum and the canonical momentum. The total momentum is conserved, whichever momentum we use, and this leads us to identify, unambiguously, the Abraham and Minkowski momenta, respectively, as the kinetic and canonical momenta for the light.

Finally, we note that a number of momenta have been proposed as rivals to those of Abraham and Minkowski (Brevik 1979), with the aim of thereby resolving the conflict. We may hope that in demonstrating, clearly, the need for and natures of both the Abraham *and* Minkowski momenta, we may also have removed the need for these and further rival momenta.
